# Effects of infant touch on the growth and neurodevelopment of preterm infants

**DOI:** 10.12669/pjms.42.2.13094

**Published:** 2026-02

**Authors:** Qingqing Yang, Qiuying Du, Huichan Yang, Yanfei Ma, Hongbin Zhu

**Affiliations:** 1 Qingqing Yang Department of Paediatrics, Maternity & Child Care Center of Qinhuangdao, Qinhuangdao 066000, Hebei, China; 2 Qiuying Du Department of Paediatrics, Maternity & Child Care Center of Qinhuangdao, Qinhuangdao 066000, Hebei, China; 3 Huichan Yang Department of Paediatrics, Maternity & Child Care Center of Qinhuangdao, Qinhuangdao 066000, Hebei, China; 4 Yanfei Ma Department of Paediatrics, Maternity & Child Care Center of Qinhuangdao, Qinhuangdao 066000, Hebei, China; 5 Hongbin Zhu Department of Paediatrics, Maternity & Child Care Center of Qinhuangdao, Qinhuangdao 066000, Hebei, China

**Keywords:** Anxiety and depression, Growth, Infant touch, Neurodevelopment, Preterm infants

## Abstract

**Objective:**

To investigate the effects of infant touch on the growth and neurodevelopment of preterm infants.

**Methodology:**

An observational study was conducted involving 64 preterm infants and their mothers, who were recruited from the Maternity & Child Care Centre of Qinhuangdao between April 2023 to May 2024. They were randomly assigned to either the control or the touch group. The control group received conventional care, whereas the touch group received infant touch therapy in addition to conventional care. The two groups were compared in terms of growth parameters, neurodevelopmental outcomes and maternal emotional well-being. Growth indices, neurodevelopmental scores and maternal emotional scores were assessed and analysed.

**Results:**

On the first day of life, no statistically significant differences were observed between the groups for any infant or maternal variables (*P* > 0.05). After 35 days, the touch group demonstrated significantly greater body weight, length, head circumference and milk intake than the control group. Neuro-behavioural scale scores were also higher in the touch group. In addition, maternal depression and anxiety scores were lower in the touch group, whereas maternal satisfaction scores were higher and all differences reached statistical significance (*P* < 0.05).

**Conclusion:**

Infant touch is an effective intervention for promoting growth in preterm infants, while alleviating maternal anxiety and depression, exerting a positive impact on the growth and neurodevelopment of preterm infants.

## INTRODUCTION

Preterm infants are defined as those born alive before the completion of 37 weeks of gestation. Globally, approximately 30 million infants are born preterm each year.^1^ Owing to incomplete organ development, preterm infants are vulnerable to short- and long-term adverse effects on growth and neurodevelopment. These effects may include considerable deficits in height and weight gain, cerebral palsy, chronic lung disease, visual and hearing impairments, attention deficits, coordination disorders and in severe cases, death, if exposed prematurely to harmful stimuli.^2^,^3^ Hence, preterm birth and its associated health risks have emerged as a global public health concern.^4^ The tactile sense is the most developed sensory modality in infants at birth and plays an essential role in their growth and developmental processes. Through touch, infants perceive their environment and establish early communication with their parents.^5^,^6^ Infant touch, a structured form of tactile stimulation, has been shown to foster trust in preterm infants, promote weight gain, stimulate bone growth and enhance neurodevelopment.^7^

Mothers of preterm infants are susceptible to postpartum anxiety due to concerns regarding their infants’ survival, the risk of complications and a lack of specialised nursing skills.^8^ Infant touch can alleviate maternal anxiety and depression while enhancing maternal self-confidence.^9^ However, opportunities for such contact are often restricted because preterm infants require frequent admission to the neonatal intensive care unit (NICU).^10^-^12^ Accordingly, healthcare teams should implement strategies to improve maternal touch skills, increase the frequency and duration of mother-infant contact and expand the provision of professional touch therapy by nurses. Such measures can promote preterm infant growth and neurodevelopment, reduce maternal anxiety and depression and improve maternal satisfaction. The present study was therefore designed to evaluate the effects of touch massage administered by mothers in conjunction with healthcare team interventions on the growth and neurodevelopment of preterm infants.

## METHODOLOGY

This observational study was conducted at the Maternity & Child Care Centre of Qinhuangdao between April 2023 to May 2024. A total of 64 preterm infants and their mothers were enrolled and randomly assigned to one of two groups based on nursing care: the control (n = 32) and touch groups (n = 32). The control group included 18 males and 14 females with a mean gestational age of 34.12 ± 1.40 weeks or a mean maternal age of 26.66 ± 3.66 years. The touch group was composed of 17 males and 15 females with a mean gestational age of 34.147 ± 1.24 weeks or a mean maternal age of 27.47 ± 3.56 years. No statistically significant differences were observed in the baseline characteristics of the infants or mothers between the two groups (*P* > 0.05).

### Ethical Approval:

The study was approved by the Institutional Ethics Committee of Maternity & Child Care Centre of Qinhuangdao (approval no. QHDFY-2023041910; date: 19 April 2023). Written informed consent was obtained from all legal guardians prior to participation.

### Inclusion criteria:

- Preterm infants born between 30 and 36 weeks of gestational age.
- Preterm infants in good general condition, without severe comorbidities or gastrointestinal abnormalities.
- Birth weight of less than 2.5 kg.
- Apgar score of ≥8 at 1 and 5 minutes after birth.
- Age of 22-32 years, being primiparous and the ability to communicate normally.
- Voluntarily participation and written informed consent.

### Exclusion criteria:

- Mothers with birth-related injuries or serious infectious diseases, such as AIDS and syphilis.
- Preterm infants with severe medical conditions, including hereditary metabolic disorders, congenital heart disease, sepsis and congenital malformations.
- Preterm infants with skin integrity issues (e.g., open wounds).

### Methods Control group:

Infants in the control group received routine neonatal care.

- Body temperature was stabilised by maintaining the room temperature at approximately 22 °C, the relative humidity at approximately 55%-60% and adequate ventilation. Infant body temperature was kept within 36-37 °C and the frequency of opening and closing the incubator was minimized to reduce fluctuations in temperature and humidity.
- Vital signs, including temperature, respiration and pulse, were monitored four times daily and any abnormality was promptly reported to physicians for appropriate management.
- To prevent infection, a disinfection and isolation protocol was implemented, requiring healthcare workers to wash and disinfect their hands before providing care.
- Respiratory function was maintained by ensuring airway patency and promptly clearing intranasal secretions.
- Skin care included daily bathing and the application of diaper cream or anti-rash powder as appropriate.
- Nutritional needs were addressed through scientifically guided dietary management.

### Touch group:

In addition to the routine neonatal care described in section, infants in the touch group received infant touch therapy. Appropriate times for intervention were selected and the room temperature was increased by 1-2 °C prior to the procedure. Touch providers were required to wash and disinfect their hands and ensure the cleanliness of the operating environment. The touch sequence followed a standardised order: head, chest, abdomen, upper limbs, lower limbs, back and buttocks. Each area was gently stimulated four to six times with consistent, coherent and skillful movements at an appropriate intensity. Sessions were conducted twice daily, lasting 10-15 minutes each and were discontinued if the infant appeared satisfied or exhibited distress, such as persistent crying. To ensure proper technique, one educational lecture on infant touch was delivered to mothers or family members, followed by three individualised hands-on training sessions. After hospital discharge, mothers or family members continued providing touch therapy at home and weekly follow-up telephone calls were made to ensure the accuracy of study data.

### Blinding Procedure:

### Parental non-blinded design:

Due to the nature of infant touch therapy intervention (requiring active parental participation in skin contact), this study is a non-blinded study. The parents were aware of the grouping situation but were told to avoid communicating the details of the intervention with other subjects.

### Evaluators were blinded:

Neurodevelopmental assessment (NBNA) was performed by neonatologists who were unaware of the grouping situation, and standardized scoring scales were used to reduce measurement bias.

### Statistical analysts are blinded:

In the data analysis stage, the blinding method is adopted, and statistical personnel only come into contact with anonymous grouping codes.

### Evaluation indexes:

### Comparison of growth indexes:

Body weight, length, head circumference and milk intake of preterm infants were measured as indicators of growth and higher values reflect better growth outcomes.

### Comparison of neurodevelopmental scores:

Neurodevelopment was assessed using the Neonatal Behavioural Neurological Assessment (NBNA), which includes five domains: primitive reflexes, passive muscle tone, behavioural capacity, general reactions and active muscle tone. Each domain is scored out of 10 and higher scores indicate better neurodevelopment.

### Comparison of maternal emotional scores:

Maternal emotional status was evaluated using the Self-Rating Depression Scale (SDS) and the Self-Rating Anxiety Scale (SAS). On the SAS, a score of 50 or above indicated anxiety, whereas on the SDS, a score of 53 or above indicated depressive symptoms. In both scales, higher scores represented greater severity. Maternal satisfaction was measured using the Nursing Service Satisfaction Scale (NSNS), developed with reference to the literature from the China National Knowledge Infrastructure database. The NSNS consists of 20 items, each scored from 1 point to 5 points for a total score of 100 and higher scores indicate greater satisfaction.

### Statistical analysis:

All data were analysed using SPSS version 22.0 (IBM Corp., Armonk, NY, USA). The sample size was denoted by *n*. Measurement data were expressed as mean ± standard deviation (*x̄* ± *s*). A *P* value of<0.05 was considered indicative of statistical significance. Comparisons between groups were performed using the independent-samples *t*-test.

## RESULTS

No statistically significant difference was found between the two groups at baseline in terms of body weight, length, head circumference or milk intake (*P* > 0.05). as shown in Table-I. After 35 days of intervention, the touch group demonstrated significantly greater body weight, length, head circumference and milk intake than the control group (*P* < 0.05).

**Table-I:** Comparison of growth indexes (N=32, *χ̅*±*S*).

Group	Days (d)	Touch group	Control group	t	P
Body weight (g)	1	2050.94±217.65	2045.03±213.58	0.110	0.913
	35	3399.09±234.51	3238.38±129.15	3.396	0.001
Length (cm)	1	45.20±2.90	45.31±2.79	-0.153	0.879
	35	52.59±3.32	47.44±2.12	7.400	1.0525E-9
Head circumference (cm)	1	32.75±2.34	32.76±2.45	-0.020	0.984
	35	36.28±2.99	34.47±1.22	3.179	0.003
Milk intake (mL)	1	217.53±25.47	224.31±25.01	-1.075	0.287
	35	510.47±28.27	444.47±17.28	11.269	1.723E-15

No statistically significant differences between the groups on the first day of life in primary reflexes, passive muscle tone, behavioural capacity, general reactions or active muscle tone (*P* > 0.05). Table-II. After 35 days of care, all neurodevelopmental indices in the touch group were significantly higher than those in the control group (*P* < 0.05).

**Table-II:** Comparison of NBNA scores (N=32, *χ̅*±*S*, points).

Group	Days/d	Touch group	Control group	t	P
Primary reflexes	1	3.77±0.09	3.77±0.10	-0.257	0.798
	35	6.59±0.16	5.50±0.26	21.910	2.119E-27
Passive dystonia	1	4.20±0.19	4.16±0.17	0.794	0.430
	35	7.13±0.20	5.80±0.81	9.059	1.088E-10
Behavioral ability	1	8.67±0.46	8.68±0.50	-0.057	0.955
	35	14.83±1.10	13.16±0.71	7.232	1.896E-9
General response	1	2.42±0.29	2.47±0.35	-0.632	0.530
	35	8.15±0.48	7.26±0.23	9.496	2.989E-12
Active dystonia	1	5.09±0.14	5.04±0.25	0.925	0.359
	35	8.04±0.76	5.97±0.51	12.838	4.088E-18

Both groups of mothers exhibited varying degrees of depressive symptoms 35 days after preterm birth. Table-III However, the touch group showed significantly lower anxiety and depression scores and significantly higher satisfaction scores compared with the control group (*P* < 0.05).

**Table-III:** Comparison of maternal emotional scores (N=32, *χ̅*±*S*, points).

Group	SDS score	SAS score	NSNS score
Touch group	52.34±3.96	33.07±7.05	96.61±1.39
Control group	58.47±5.86	44.67±2.86	88.83±2.45
*t*	-4.898	-8.623	15.606
*P*	9.000E-6	9.519E-11	1.165E-20

## DISCUSSION

The findings of this study indicate that infant touch, administered over 35 days, significantly improved growth parameters (body weight, length, head circumference, and milk intake) and early neurodevelopmental scores (as measured by the NBNA) in preterm infants, while also reducing maternal anxiety and depression and increasing maternal satisfaction. These results underscore the potential of infant touch as a simple, low-cost intervention in the early care of preterm infants.

Studies have shown that the incidence of preterm birth in China has increased from 5.9% in 2012 to 6.4% in 2018.^13^ Preterm infants are susceptible to complications, including growth retardation, respiratory distress syndrome, bronchopulmonary dysplasia, necrotising enterocolitis, neurodevelopmental disorders, chronic lung disease, hypertension and glucose intolerance. These conditions are the leading causes of mortality in new-borns and children under five years of age.^14^ Hence, early interventional nursing care should be implemented to reduce the incidence and mitigate the impact of complications in preterm infants.^15^,^16^

In line with previous research, our study confirms that infant touch promotes weight gain and growth in preterm infants. As a natural low-cost intervention, infant touch features simplicity and affordability, reducing the economic burden of caring for preterm infants and alleviating stress on families. By stimulating the parasympathetic nervous system, infant touch enhances gastric peristalsis and insulin secretion, reduces the incidence of gastric retention and abdominal distension and supports healthy weight gain. Similar effects on growth have been reported in studies conducted in various settings.^17^,^18^ In the present study, after 35 days of infant touch care, the milk intake of the touch group was higher than that of the control group. The resulting increase in protein and nutrient intake likely contributed to the accelerated growth of preterm infants. Consequently, the body weight, length and head circumference of infants in the touch group were significantly greater than those in the control group (*P* < 0.05).

Our neurodevelopmental findings also align with existing evidence that tactile stimulation can support early brain development. Neurological development in infants occurs primarily during the second trimester of gestation. Owing to incomplete brain maturation, preterm infants are susceptible to adverse environmental factors in the NICU, such as bright light, noise and procedural pain.^19^ Approximately 20% of preterm infants continue to experience neurodevelopmental delays by the age of 10 years.^20^ Appropriate infant touch stimulates the parasympathetic activity, promotes cortical and structural brain development and accelerates the rate of neurodevelopment in preterm infants. This aligns with broader evidence highlighting the importance of sensory-based, developmental care in supporting preterm brain maturation and behavioral organization.^6^,^19^ In the present study, neurodevelopmental outcomes were evaluated using NBNA scores. Although both groups demonstrated improvements after 35 days of care, the touch group achieved higher scores in primitive reflexes, passive muscle tone, behavioural capacity, general reactions and active muscle tone than the control group (P < 0.05), exhibiting a faster rate of neurodevelopment.

Importantly, our study reinforces the bidirectional benefit of infant touch, extending positive effects to maternal psychological well-being. This aligns with findings that maternal involvement in infant care can improve maternal confidence and emotional state.^8^ Research on infant touch remains limited and most studies have focused primarily on its unidirectional effects on the development of preterm infants.^21^ Infant touch exerts bidirectional effects on preterm infants and their mothers. By increasing the duration of physical contact, infant touch fosters emotional communication between mother and infant, which not only supports neonatal development but also reduces maternal anxiety and depression. In the present study, mothers in the touch group received professional training in touch techniques, which enhanced their confidence in caring for their infants. The increased milk intake observed in the preterm infants of the touch group likely stimulated maternal lactation, creating a positive feedback cycle that benefitted mother and infant. Consistent with these effects, the touch group demonstrated significantly lower depression and anxiety scores and significantly higher maternal satisfaction scores compared with the control group (*P* < 0.05).

### Generalizability and Limitations:

While our results are encouraging, their generalizability may be influenced by several factors. This study has certain limitations. The single-center design and relatively small sample size may restrict the generalizability of the findings. The results are most directly applicable to stable preterm infants (30–36 weeks gestational age) in secondary or tertiary care settings in urban China. Further research is needed to determine whether similar benefits would be observed in more medically complex preterm infants, in different cultural contexts, or in resource-limited settings where nursing ratios and family involvement patterns may differ. The absence of long-term neurodevelopmental follow-up limits conclusions about sustained effects, and the reliance on the NBNA as the sole neurodevelopmental assessment tool, despite its validity for early evaluation, indicates a need for complementary standardized measures in future research to provide a more comprehensive developmental profile. Although maternal and family involvement was enhanced, infant touch requires considerable time. Future research should explore strategies to reduce this burden while maintaining the developmental benefits for infants. Future studies should also consider larger, multicenter cohorts with extended follow-up to validate and expand upon these findings. Routine clinical monitoring (without formal NBNA) continued through three months. This design choice prioritized detecting initial neurological effects while minimizing assessment burden. Potential approaches include incorporating audio-recorded accompaniment for mothers, facilitating mother-infant clothing exchanges to enhance bonding and increasing the involvement of specialised nurses in providing touch therapy.

## CONCLUSIONS

Infant touch is a valuable adjunct to conventional neonatal care, effectively enhancing growth and neurodevelopment in preterm infants while reducing maternal anxiety and depression.

### Authors’ Contributions:

**QY & QD:** Manuscript writing, and is responsible for the integrity of the study.

**HY:** Collected the data and performed the analysis.

**YM** and **HZ:** Conceived and designed the study. Critical Review.

All authors have read and approved the final manuscript.

## References

[1] Garnica-Torres Z, A G, Pedroso J D S (2021). Attachment between father and premature baby in kangaroo care in a neonatal unit of a public hospital. J Neonatal Nurs.

[2] Lalanne J, Leduc-Pessah H, Buba M, Reisman J (2023). When It Is Not BPD, What Could It Be?A Preterm Infant with Persistent Tachypnea and Increased Work of Breathing. Clin Pediatr (Phila).

[3] Fahimuddin FZ, Etemadi K, Morgan B (2022). A Quiescent Infection in a Premature Newborn. Clin Pediatr (Phila).

[4] Chawanpaiboon S, Vogel JP, Moller AB, Lumbiganon P, Petzold M, Hogan D (2019). Global, regional and national estimates of levels of preterm birth in 2014: a systematic review and modelling analysis. Lancet Glob Health.

[5] Croy I, Mohr T, Weidner K, Hummel T, Junge-Hoffmeister J (2019). Mother-child bonding is associated with the maternal perception of the child's body odor. Physiol Behav.

[6] Bear RJ, Mellor DJ (2017). Continuing Education Module-Kangaroo Mother Care 2: Potential Beneficial Impacts on Brain Development in Premature Infants. J Perinat Educ.

[7] Fadlalmola HA, Elhusein AM, Abedelwahed HH, Mohmed SA, Alnassry SMA, Mohammed FH (2023). The effects of Yakson touch and gentle human touch on preterm infants: A systematic review and meta-analysis. Afr J Reprod Health.

[8] Mercuri M, Stack DM, Mantis I, Moszkowski R, Field TM (2023). Maternal and infant touching behaviours during perturbed interactions: Associations with maternal depressive symptomatology and infant crying. Infant Behav Dev.

[9] Siva N, Nayak BS, Lewis LES, Velayudhan B, Phagdol T, Sathish Y (2023). Involvement of mothers in high-risk neonatal care: A capacity building program for neonatal nurses. J Neonatal Nurs.

[10] Hara T, Kasahara Y, Nakagawa T (2022). Pre-dialysis diastolic blood pressure and intradialytic hypotension in patients undergoing maintenance haemodialysis. J Nephrol.

[11] Li G, Wang L, Yap PT, Wang F, Wu Z, Meng Y (2019). Computational neuroanatomy of baby brains: A review. Neuroimage.

[12] Karimi FZ, Miri HH, Khadivzadeh T, Maleki-Saghooni N (2020). The effect of mother-infant skin-to-skin contact immediately after birth on exclusive breastfeeding: a systematic review and meta-analysis. J Turk Ger Gynecol Assoc.

[13] Deng K, Liang J, Mu Y, Liu Z, Wang Y, Li M (2021). Preterm births in China between 2012 and 2018: an observational study of more than 9 million women. Lancet Glob Health.

[14] Purisch SE, Gyamfi-Bannerman C (2017). Epidemiology of preterm birth. Semin Perinatol.

[15] Pickler RH, Meinzen-Derr J, Moore M, Sealschott S, Tepe K (2020). Effect of Tactile Experience During Preterm Infant Feeding on Clinical Outcomes. Nurs Res.

[16] Rad ZA, Aziznejadroshan P, Amiri AS, Ahangar HG, Valizadehchari Z (2021). The effect of inhaling mother's breast milk odor on the behavioral responses to pain caused by hepatitis B vaccine in preterm infants: a randomized clinical trial. BMC Pediatr.

[17] Dorum BA, Ozkan H, Cakir SC, Koksal N, Sen GE (2019). What should be the protein target for adjustable Human Milk fortification in premature infants?. Pak J Med Sci.

[18] Du Q, Zhu H, Guo M, Yang Q, Yang H, Ma Y (2024). The effect of baby touch on premature infants. Pak J Med Sci.

[19] Khoramirad A, Taheri L, Eskandari N, Abedini Z (2023). Effect of mindfulness-based neurodevelopmental care on infant outcomes in NICU[J/OL. J Neonatal Nurs.

[20] Johnston KM, Gooch K, Korol E, Vo P, Eyawo O, Bradt P (2014). The economic burden of prematurity in Canada. BMC Pediatr.

[21] Widström AM, Brimdyr K, Svensson K, Cadwell K, Nissen E (2020). A plausible pathway of imprinted behaviors: Skin-to-skin actions of the newborn immediately after birth follow the order of fetal development and intrauterine training of movements. Med Hypotheses.

